# SUMOylation of PUM2 promotes the vasculogenic mimicry of glioma cells via regulating CEBPD

**DOI:** 10.1002/ctm2.168

**Published:** 2020-09-06

**Authors:** Di Wang, Xuelei Ruan, Xiaobai Liu, Yixue Xue, Lianqi Shao, Chunqing Yang, Lu Zhu, Yang Yang, Zhen Li, Bo Yu, Tianda Feng, Yunhui Liu

**Affiliations:** ^1^ Department of Neurosurgery Shengjing Hospital of China Medical University Shenyang China; ^2^ Liaoning Clinical Medical Research Center in Nervous System Disease Shenyang China; ^3^ Key Laboratory of Neuro‐oncology in Liaoning Province Shenyang China; ^4^ Department of Neurobiology, School of life Sciences China Medical University Shenyang China; ^5^ Key Laboratory of Cell Biology, Ministry of Public Health of China China Medical University Shenyang China; ^6^ Key Laboratory of Medical Cell Biology, Ministry of Education of China China Medical University Shenyang China

**Keywords:** CEBPD, PUM2, SUMOylation, UBE2I, vasculogenic mimicry

## Abstract

Glioma is the most common form of primary central nervous malignant tumors. Vasculogenic mimicry (VM) is a blood supply channel that is different from endothelial blood vessels in glioma. VM is related to tumor invasion and metastasis. Therefore, it plays an important role to target therapy for glioma VM. Our experimental results showed abnormal expression of UBE2I, PUM2, CEBPD, and DSG2 in glioma cells. The Co‐IP and Immunofluorescence staining were used to detect that PUM2 can be modified by SUMO2/3. The interaction between PUM2 and CEBPD mRNA was detected by the RIP assays. The interaction between transcription factor CEBPD and promoter region of DSG2 was detected by the ChIP assays and luciferase assays. The capacity for migration in glioma cells was observed by the laser holographic microscope. The capacity for invasion in glioma cells was detected by Transwell method. The VM in glioma cells was detected by three‐dimensional cell culture method. The experimental results found that the upregulation of UBE2I in glioma tissues and cells promotes the SUMOylation of PUM2, which decreases not only the stability of PUM2 protein but also decreases the inhibitory effect of PUM2 on CEBPD mRNA. The upregulation of CEBPD promotes the binding to the upstream promoter region of DSG2 gene, further upregulates the expression of DSG2, and finally promotes the development of glioma VM. In conclusion, this study found that the UBE2I/PUM2/CEBPD/DSG2 played crucial roles in regulating glioma VM. It also provides potential targets and alternative strategies for combined treatment of glioma.

## Graphical abstract

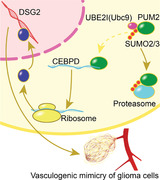

## 1 INTRODUCTION

In the nervous system, glioma is the most common primary tumor, and its incidence accounts for about 40% of primary intracranial tumors. The invasive growth of the tumor makes it difficult to treat, and the tumor is prone to recur. Therefore, the mortality rate of patients is extremely high.^1^ In order to improve the curative effect on gliomas, more and more researchers are committed to developing new therapeutic drugs for molecular targets, including tumor markers, abnormal signaling pathways, epigenetic gene expression regulation, and tumor vascular growth inhibitors and tumor immunotherapy.^2^, ^3^, ^4^ Currently, the treatment of antitumor angiogenesis has become one of the hot spots of glioma research, but in clinical applications, antiangiogenic drugs represented by bevacizumab have been far from the expected efficacy.^5^ Vasculogenic mimicry (VM) phenomenon is a model of tumor microcirculation that does not depend on endothelial cells,^6^, ^7^ which refers to the tube structure formed by tumor cells with “endothelial cell function” through self‐deformation and matrix remodeling. Such tumor cells exhibit multiple phenotypes, such as dedifferentiation and embryogenesis, and have the dual characteristics of “endothelial cells” and “tumor cells.” Many studies have shown that VM exists in many fast‐growing solid tumors, like hepatocellular carcinoma, non‐small cell lung cancer, lung adenocarcinoma, and breast cancer.^8^, ^9^, ^10^, ^11^ The existence of VM greatly decreases the efficacy of antiangiogenesis in chemotherapy drugs.^12^ VM in glioma is closely related to the malignant degree of glioma.^13^ Therefore, further research on the molecular mechanism of glioma VM has important significance to find a more effective treatment for glioma.

The small ubiquitin‐like modifier (SUMO) includes four members of SUMO1, SUMO2, SUMO3, and SUMO4, a class of small proteins with a molecular weight of about 10 KDa, which can be reversibly bound to the substrate protein by a covalent bond. The above modification process is called SUMOylation that is catalyzed by an enzymatic cascade. This enzymatic reaction involves four essential catalytic enzymes, including the activating enzyme (E1), the conjugating enzyme (E2, Ubc9 is the only known conjugating enzyme, encoded by UBE2I gene), the ligase (E3), and specific proteases that can reverse SUMOylation (SENPs, SENP3 is the one of the most widely studied).^14^ SUMOylation of the cells involved in transcriptional regulation, nuclear and cytoplasmic transport, the adjustment process to maintain genomic stability, and is closely related to the development of various tumors.^15^, ^16^, ^17^ The deletion of SENP3 increases the SUMO3 modification at the K^310^ amino acid position of IRF8, upregulates the expression of NFATc1 and osteoclastogenesis, and ultimately leads to osteoporosis in mice.^18^ For aggressive pancreatic ductal adenocarcinoma, SUMO inhibitors can be used as targeted therapeutic drugs.^19^ In gliomas, the SUMOylation of CDK6 inhibits ubiquitin‐mediated degradation of CDK6, stabilizes CDK6 protein, regulates the cell cycle, and ultimately drives the development of gliomas.^20^ In prostate cancer DU145 cells, the SUMO1 modification of KHSRP can promote the VM of the cell by preventing the extension of the short G‐rich region in the pre‐miRNA terminal loop.^21^

Pumilio RNA Binding Family Member 2 (PUM2) is a member of the PUF family of sequence‐specific RNA binding proteins. Members of this family can bind to the target mRNA network containing pumilio response elements (PRE/PBE), and inhibit the expression of target proteins by showing translation and promoting mRNA decay.^22^ In human dermal keratinocytes, PUM2 can mediate cells inflammation and apoptosis through the AuroraA/NF‐κB pathway.^23^ The high expression of PUM2 in breast cancer tissues is inversely related to the overall survival and recurrence‐free survival of breast cancer patients.^24^ In osteosarcoma, the expression of PUM2 is low, and the overexpression of PUM2 inhibits the dryness of osteosarcoma.^25^

CCAAT Enhancer Binding Protein Delta (CEBPD), as a b‐ZIP transcription factor, can bind DNA regulatory regions to promote transcription. It plays an important role in regulating immune and inflammatory responses.^26^ CEBPD expression is significantly increased in inflammatory diseases, such as Alzheimer's disease and rheumatoid arthritis.^27^, ^28^ It is involved in the process of antioxidative stress in astrocytes.^29^ Activated CEBPD can promote the activity of chemical inducers and the migration of microglia/macrophages by activating MCP‐1 and MMPs.^30^ In bladder urothelial carcinoma, gefitinib treatment significantly decreases the expression of CEBPD and enhances the sensitivity of bladder urothelial cancer cells to cisplatin and paclitaxel.^31^ In hepatocellular carcinoma and breast cancer, CEBPD regulates the biological behavior of tumor cells.^32^, ^33^

The Desmoglein 2 (DSG2) gene is located at 18q12.1, and the encoded protein is a calcium‐dependent desmosomal cadherin, which is abnormally expressed in a variety of tumor tissues, such as breast cancer, squamous cell carcinoma, and non‐small cell lung cancer. It is involved in regulating tumor cell proliferation, cell cycle regulation, and tumor metastasis.^34^, ^35^, ^36^ Recent studies have shown that the expression of DSG2 in human melanoma is associated with VM.^37^ However, the role of SUMO2/3, PUM2, CEBPD, and DSG2 in glioma VM has not been reported.

The purpose of this study is to clarify the SUMO2/3‐induced SUMOylation in glioma, as well as the expression and interaction of PUM2, CEBPD, and DSG2, and the role of these molecules in regulating VM of glioma. It is hoped that the results of this study can provide a new breakthrough point for the formation of glioma VM.

## 2 MATERIALS AND METHODS

### 2.1 Pathological tissue acquisition and cell culture

The patient's human glioma tissue and normal brain tissue were collected from the Department of Neurosurgery, Shengjing Hospital, China Medical University. All patients voluntarily signed an informed consent form. The study was approved by the ethics committee of Shengjing Hospital affiliated to China Medical University. HEK‐293T (human embryonic kidney) cells, U251 cells, and U373 cells (Human glioma cells) were purchased from Shanghai Gene Chemistry Co., Ltd. The human astrocytes (HA) cells used in the study were purchased from Shanghai Zeye Biotechnology. See online Additional Materials and Methods for details of storage and cell culture methods.

### 2.2 Quantitative real‐time PCR

The RNA expression of various indicators in the study was detected by quantitative real‐time PCR (qRT‐PCR) method. Trizol reagent (Life Technologies Corporation, Carlsbad, CA, USA) was used to extract RNA from various tissues and cells. The 7500 Fast RTPCR System is used to quantitatively analyze the RNA expression of various indicators in the study. For details of the experiment, please refer to online Additional Materials and Methods.

### 2.3 Cell transfection

The overexpression plasmids, knockdown plasmids, and mutant plasmids of various indicators in this study were purchased from Gene‐Pharama (Shanghai, China) and JTS (Beijing, China). Resistant cell clones are established by G418, Hygromycin, and Puromcin. For details of the experiment, please refer to online Additional Materials and Methods.

### 2.4 RNA binding protein immunoprecipitation assays

The RNAs bound to PUM2 were detected by RNA binding protein immunoprecipitation (RIP) assay. PUM2 protein to STARD13 mRNA as a positive control, and the IgG group as a negative control. For details of the experiment, please refer to online Additional Materials and Methods.

### 2.5 Western blot

The protein expression of various indicators in the study was detected by western blot. For details of the experiment, please refer to online Additional Materials and Methods.

### 2.6 Co‐Immunoprecipitation

Endogenous SUMOylated PUM2 was detected by co‐immunoprecipitation (Co‐IP). For details of the experiment, please refer to online Additional Materials and Methods.

### 2.7 Immunofluorescence staining

Immunofluorescence staining was used to analyze the subcellular localization and expression of the protein in this study. For details of the experiment, please refer to online Additional Materials and Methods.

### 2.8 Cell migration assays

The capacity for migration in glioma cells was observed by the HoloMonitor M4 culture system (Phase Holographic Imaging PHI AB, SE) in vitro. For details of the experiment, please refer to online Additional Materials and Methods.

### 2.9 Cell invasion assays

The capacity for invasion in glioma cells was detected by Transwell method in vitro. For details of the experiment, please refer to online Additional Materials and Methods.

### 2.10 Cells VM formation assays

The glioma cells VM was detected by three‐dimensional cell culture method with Matrigel Basement Membrane Matrix (BD Biosciences, Bedford, MA, USA). For details of the experiment, please refer to online Additional Materials and Methods.

### 2.11 Luciferase assays

The responsive CEBPD‐binding sites in the DSG2 promotor were determined by dual‐luciferase reporter system. For vectors construction and details of the experiment, please refer to online Additional Materials and Methods.

### 2.12 Chromatin immunoprecipitation assays

The responsive CEBPD‐binding sites in the DSG2 gene upstream promotor were determined by the chromatin immunoprecipitation (ChIP) Enzymatic Chromatin IP Kit (Cell Signaling Technology, Danvers, MA, USA). For details of the experiment, please refer to online Additional Materials and Methods.

### 2.13 CD34 endothelial marker periodic acid‐Schiff dual staining

CD34 endothelial marker periodic acid‐Schiff dual staining (CD34‐PAS) is used for qualitative and quantitative analyses of VM in tumor xenograft tissue sections of nude mice. For details of the experiment, please refer to online Additional Materials and Methods.

### 2.14 Tumor xenograft implantation in nude mice

The constructed stably transfected glioma cells (U251 and U373) were xenografted into immunodeficient nude mice for in vivo experiment in this study. For details of the experiment, please refer to online Additional Materials and Methods.

### 2.15 RNA‐seq analysis

The RNA‐seq data of glioma patients came from TCGA database (https://portal.gdc.cancer.gov/) and GEPIA website (http://gepia.cancer-pku.cn/).

### 2.16 Statistical analysis

The experimental data were expressed as mean ± standard deviation (SD) and were analyzed by GraphPad Prism v8.4 statistical software with the *t*‐test or one‐way ANOVA.

## 3 RESULTS

### 3.1 PUM2 is SUMOylated and degraded by binding to SUMO2/3

**FIGURE 1:**
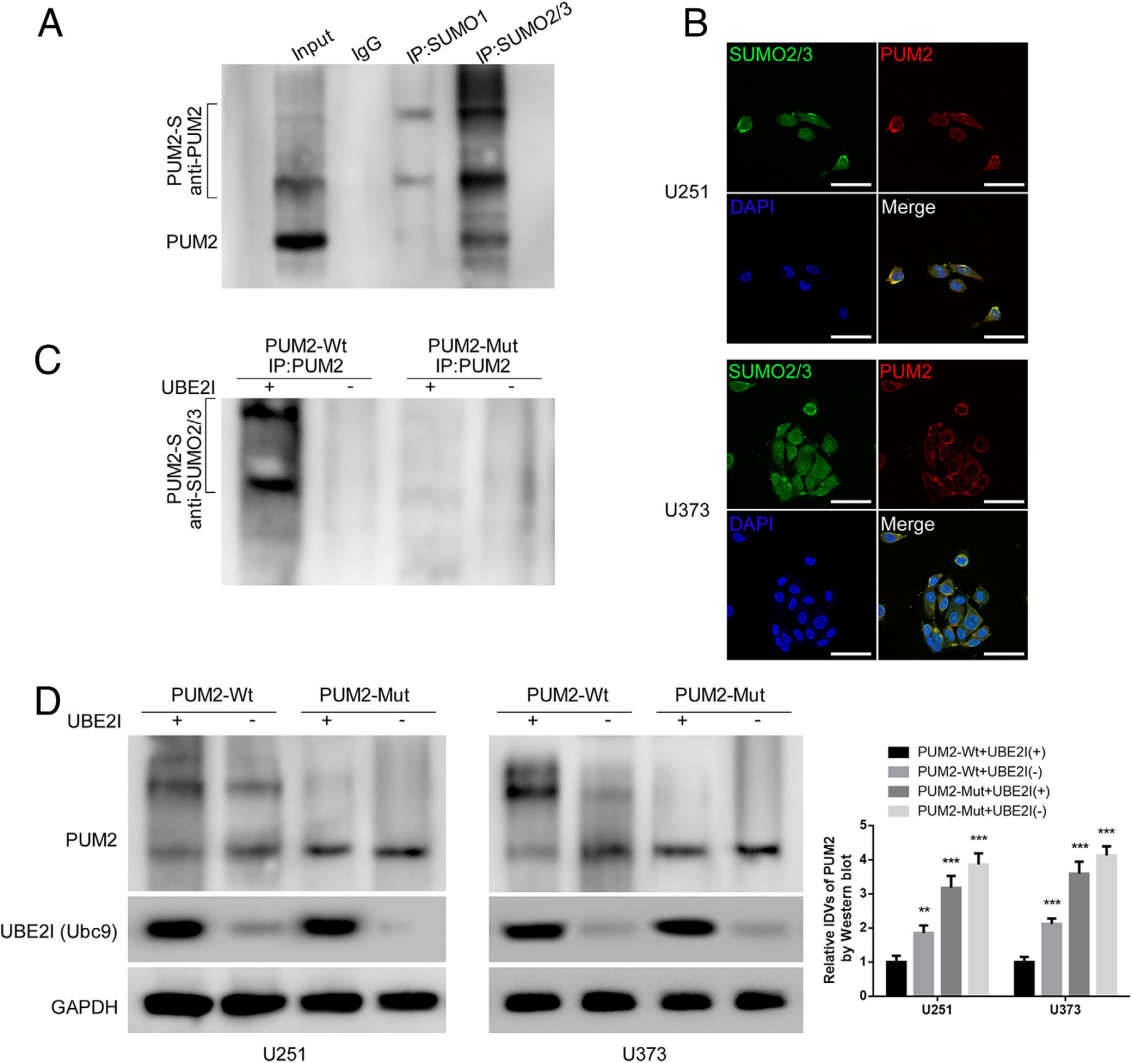
PUM2 is SUMOylated and degraded by binding to SUMO2/3:(A) PUM2 was mainly modified by SUMO2/3 in 293T cells. The Co‐IP experiment detected which SUMO protein bound to the PUM2 protein, and followed by western blot with indicated antibodies. (B) Laser scanning confocal microscopy observed the subcellular localization of PUM2 and SUMO2/3 in U251 cells and U373 cells. Scale bars: 100 µm. (C) Cotransfection of PUM2‐Wt, PUM2‐Mut (K^50/91/985^R, 3KR), and UBE2I in 293T cells, Co‐IP detected the binding of PUM2 protein and SUMO2/3, and then western blot with the indicated antibodies. (D) The western blot was used to detect the expression of PUM2 protein in U251 cells and U373 cells. Each value represents the mean±SD (n = 3), **P < .01, ***P < .001, compared with PUM2‐Wt+UBE2I(+) group

In order to investigate whether PUM2 can be SUMOylated, we first performed Co‐IP assay, and the results showed that PUM2 was mainly interacted with SUMO2/3 (Figure 1A). Based on this, we further focused on the potential modification that was caused by PUM2‐SUMO2/3. As shown in online Additional Figure 1A, Co‐IP results indicated that the binding of PUM2 and SUMO2/3 also existed in U251 and U373. By performing immunofluorescence staining under a laser scanning confocal microscopy, we found that SUMO2/3 was indistinguishably distributed in the nuclear and cytoplasm of U251 and U373, while PUM2‐SUMO2/3 complex was mainly located in cell cytoplasm (Figure 1B). Moreover, the results of western blot followed by nuclear/cytosol fractionation assay in U251 and U373 were consistent with our findings (online Additional Figure 1B). Since Ubc9 that translated by ubiquitin conjugating enzyme E2 I (UBE2I) gene was the only known SUMO‐conjugating enzyme,^38^ we wondered whether the binding between PUM2 and SUMO2/3 was mediated by Ubc9. Based on our hypothesis, we next cotransfected full‐length UBE2I plasmid and PUM2 with mutated potential binding sequence (K^50/91/985^R, 3KR) into PUM2 inhibited 293T cells. The results of Co‐IP assay confirmed the predicted SUMOylated animo acid sites on PUM2 and necessity of UBE2I in the association between PUM2 and SUMO2/3 (Figure 1C). Next, the expression of PUM2 was significantly higher in PUM2‐Mut group than in wild‐type PUM2 group, indicating that PUM2 was SUMOylated and degraded by binding to SUMO2/3 (Figure 1D). Moreover, by using proteasome inhibitor MG132, the degradation of PUM2 was significantly eliminated (online Additional Figure 1C). The above results indicated that PUM2 could bind to SUMO2/3 and be SUMOylated by proteasome.

### 3.2 UBE2I promoted the capacities for migration, invasion, and VM in glioma cells

**FIGURE 2:**
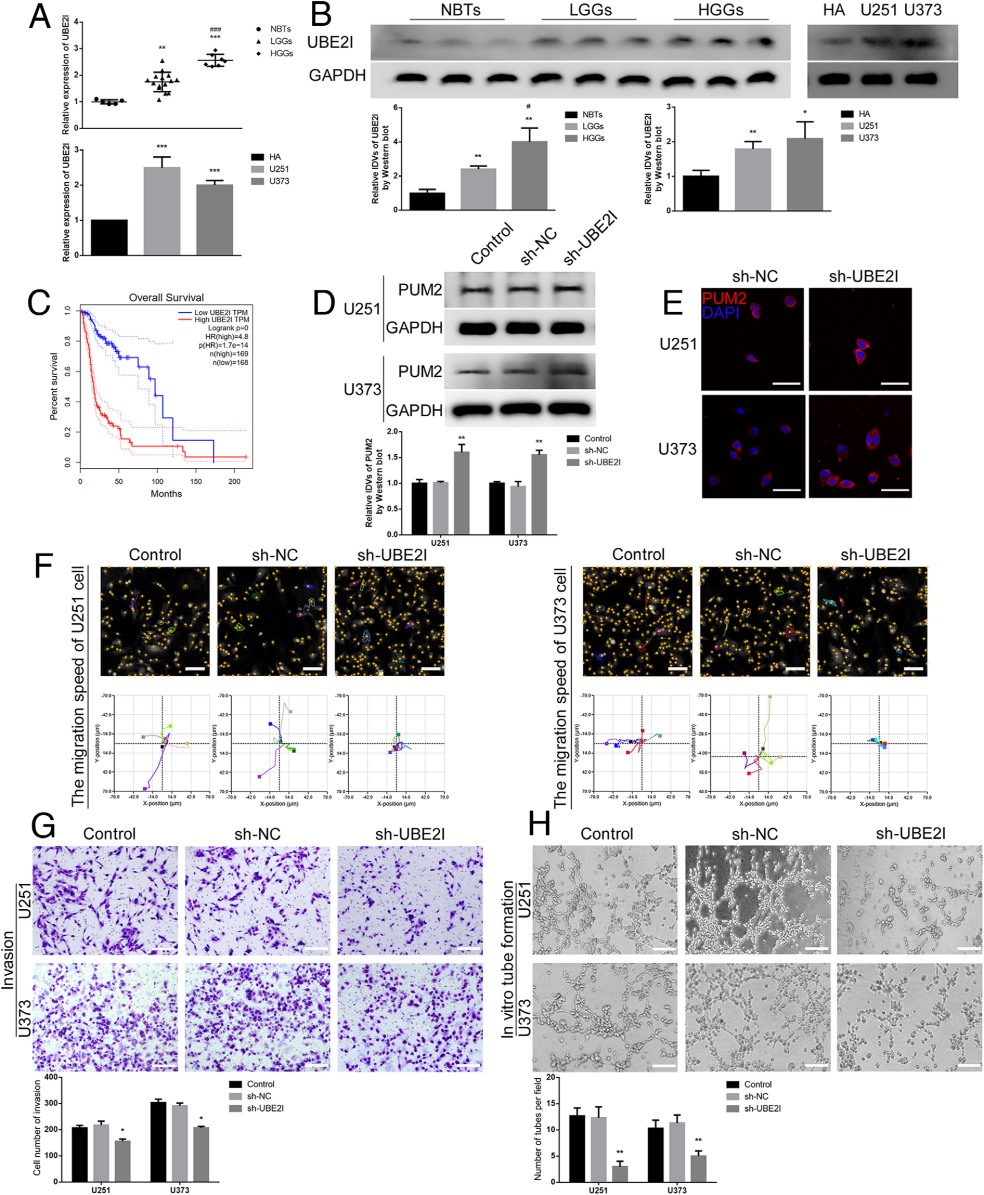
UBE2I promoted the capacities for migration, invasion, and VM in glioma cells:(A) qRT‐PCR was used to detect the expression of UBE2I in normal brain tissues (NBTs, n = 5), low‐grade gliomas (LGGs: Grade I‐II, n = 15), and high‐grade gliomas (HGGs: Grade III‐IV, n = 7) (above). Each value represents the mean±SD, **P < .01, ***P < .001, compared with NBTs group; ###*P <* .001, compared with LGGs group. qRT‐PCR was used to detect the expression of UBE2I in human astrocytes (HA) cells, U251 cells, and U373 cells (below). Each value represents the mean±SD (n = 3), **P < .01, ***P < .001, compared with HA cells. (B) Western blot was used to detect the expression of UBE2I protein in NBTs, LGGs, and HGGs (left). Each value represents the mean±SD (n = 3), **P < .01, compared with NBTs group; #*P <* .05, compared with LGGs group. Western blot was used to detect the expression of UBE2I protein in HA cells, U251 cells, and U373 cells (right). Each value represents the mean±SD (n = 3), *P < .05, **P < .01, compared with HA cells; #*P <* .05, compared with LGGs group. (C) Analysis of the expression of UBE2I in the data of glioma tissue in the TCGA database (n (high) = 169, n (low) = 168), *P <* .001. (D) Western blot was used to detect the expression of PUM2 protein in the UBE2I knockdown cells (U251 and U373). Each value represents the mean±SD (n = 3), **P < .01, compared with sh‐NC group. (E) Laser scanning confocal microscope was used to observe the subcellular changes of PUM2 protein in the UBE2I knockdown cells (U251 and U373). Scale bars: 100 µm. (F) The Hstudio M4 system observed the capacity for migration in the UBE2I knockdown cells (U251 and U373), (n = 5). Scale bars: 100 µm. (G) Transwell method was used to detect the capacity for invasion in the UBE2I knockdown cells (U251 and U373). Scale bars: 100 µm. (H) Three‐dimensional cell culture method was used to detect the change of VM in the UBE2I knockdown cells (U251 and U373). Scale bars: 200 µm. Each value represents the mean±SD (n = 3), *P < .05, **P < .01, compared with sh‐NC group

As shown in Figure 2A,B, by using qRT‐PCR and western blot, we found that the expression of UBE2I (UBC9 gene) was elevated in glioma tissues and cells, especially in high grade. By scanning TCGA database (The Cancer Genome Atlas), the expression of UBE2I was shown to be negatively correlated with the survival time of patients with low‐grade glioma and glioblastoma (Figure 2C). We further constructed U251 and U373 cells with knockdown of UBE2I, and found that compared with negative control (NC) group, knockdown of UBE2I significantly increased the expression of PUM2 (Figure 2D). Furthermore, by performing immunofluorescence staining, we noticed that the knockdown of UBE2I increased PUM2 expression in cytoplasm but did not change the cell distribution of PUM2 (Figure 2E). Finally, the invasion, migration, and VM capacities were also analyzed by Hstudio M4 software, transwell assay, and 3‐D cell culture, respectively. As shown in Figure 2F‐H, the knockdown of UBE2I significantly inhibited the capacities for migration, invasion, and VM in glioma cells, while the overexpression of UBE2I had the opposite effects (online Additional Figure 2). These results suggest that UBE2I regulates the expression of PUM2 in glioma cells, thereby further regulating the capacities for migration, invasion, and VM in glioma cells.

### 3.3 UBE2I mediated the capacities for migration, invasion, and VM in glioma cells by regulating the expression of PUM2

**FIGURE 3:**
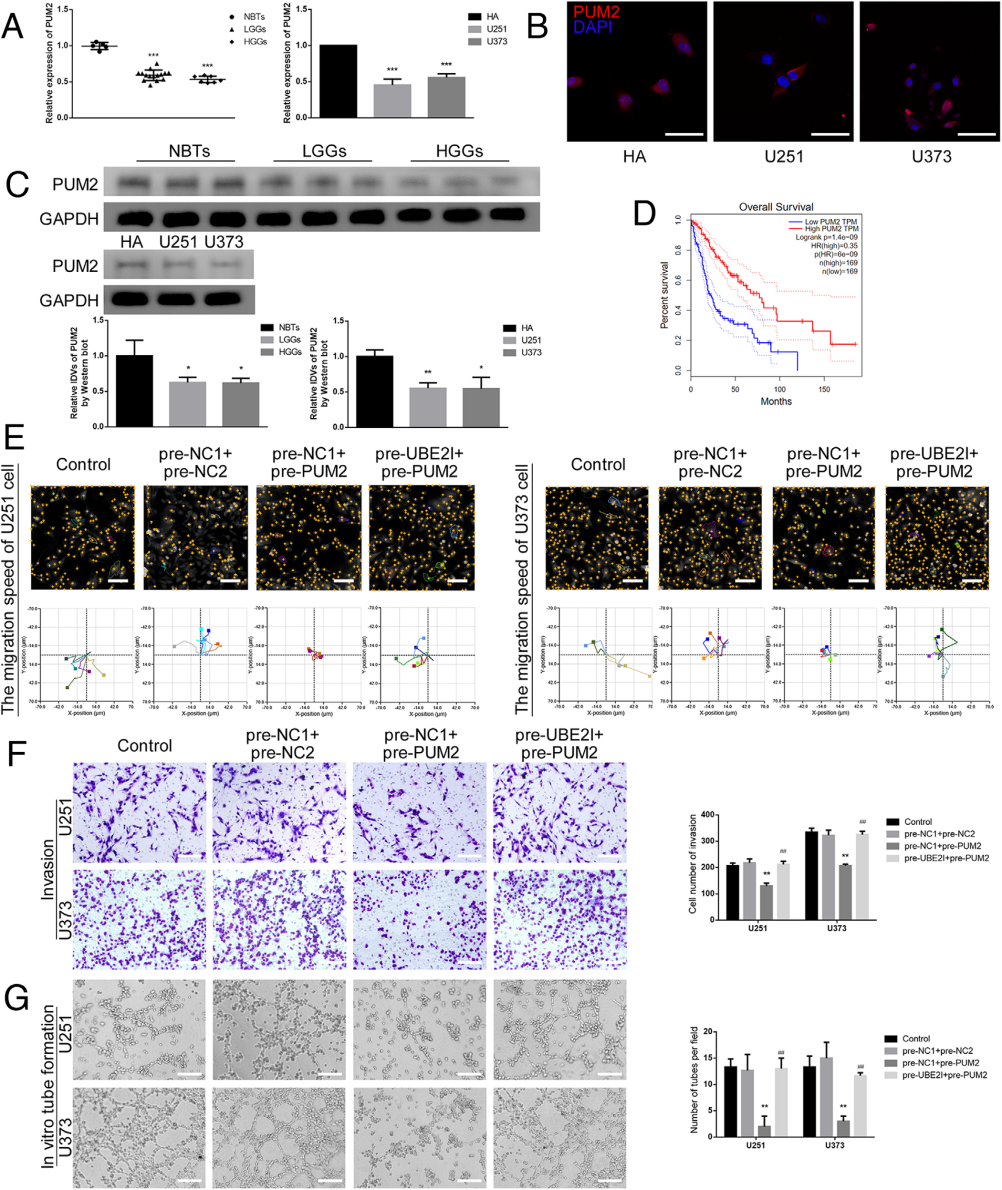
UBE2I promoted the capacities for migration, invasion, and VM in glioma cells by regulating PUM2 expression:(A) qRT‐PCR was used to detect the expression of PUM2 in NBTs (n = 5), LGGs (n = 15), and HGGs (n = 7) (left). Each value represents the mean±SD, ***P < .001, compared with NBTs group. qRT‐PCR was used to detect the expression of PUM2 in HA cells, U251 cells, and U373 cells (right). Each value represents the mean±SD, ***P < .001, compared with HA cells. (B) Immunofluorescence was used to observe the subcellular expression of PUM2 protein in HA cells, U251 cells, and U373 cells. Scale bars: 100 µm. (C) Western blot was used to detect the expression of PUM2 protein in NBTs, LGGs, and HGGs (above). Each value represents the mean±SD (n = 3), *P < .05, compared with NBTs group. Western blot was used to detect the expression of PUM2 protein in HA cells, U251 cells, and U373 cells (below). Each value represents the mean±SD (n = 3), *P < .05, **P < .01, compared with HA cells. (D) Analysis of the expression of PUM2 in the data of glioma tissue in the TCGA database (n (high) = 169, n (low) = 169), *P <* .001. (E) Hstudio M4 system was used to observe the change of the capacity for migration in the cells (U251 and U373) overexpressed UBE2I after overexpressed PUM2 (n = 5). Scale bars: 100 µm. (F) Transwell method was used to detect the capacity for invasion in cells (U251 and U373) overexpressed UBE2I after overexpressed PUM2. Scale bars: 100 µm. (G) Three‐dimensional cell culture method was used to detect the change of VM in the cells (U251 and U373) overexpressed UBE2I after overexpressed of PUM2. Scale bars: 200 µm. Each value represents the mean±SD (n = 3), **P < .01, compared with pre‐NC1+pre‐NC2 group; ##*P <* .01, compared with pre‐NC1+pre‐PUM2 group

In this study, the expression of PUM2 in the tumor tissue of glioma patients was detected by qRT‐PCR, immunofluorescence, and western blot. It was found that the expression of PUM2 in glioma tissue is significantly lower than that in normal brain tissue group, and the expression in glioma U251 and U373 cells was significantly lower than that in HA cells (Figure 3A‐C). By scanning TCGA database, it was found that the expression of PUM2 was positively correlated with the patient's survival (Figure 3D). Using the Hstudio M4 system, Transwell method, and three‐dimensional cell culture method, it was found that PUM2 overexpression significantly inhibited the capacities for migration, invasion, and VM in glioma cells compared with the NC group. Our experimental results also reported that UBE2I overexpression rescued the effect of PUM2 overexpression on the capacities for migration, invasion, and VM in glioma cells (Figure 3E‐G). Therefore, it was confirmed that UBE2I decreased the expression of PUM2 protein by SUMOylation in glioma cells, which further inhibited the capacities for migration, invasion, and VM in glioma cells.

### 3.4 PUM2 inhibited CEBPD expression by binding CEBPD mRNA, thereby inhibiting the capacities for migration, invasion, and VM in glioma cells

**FIGURE 4:**
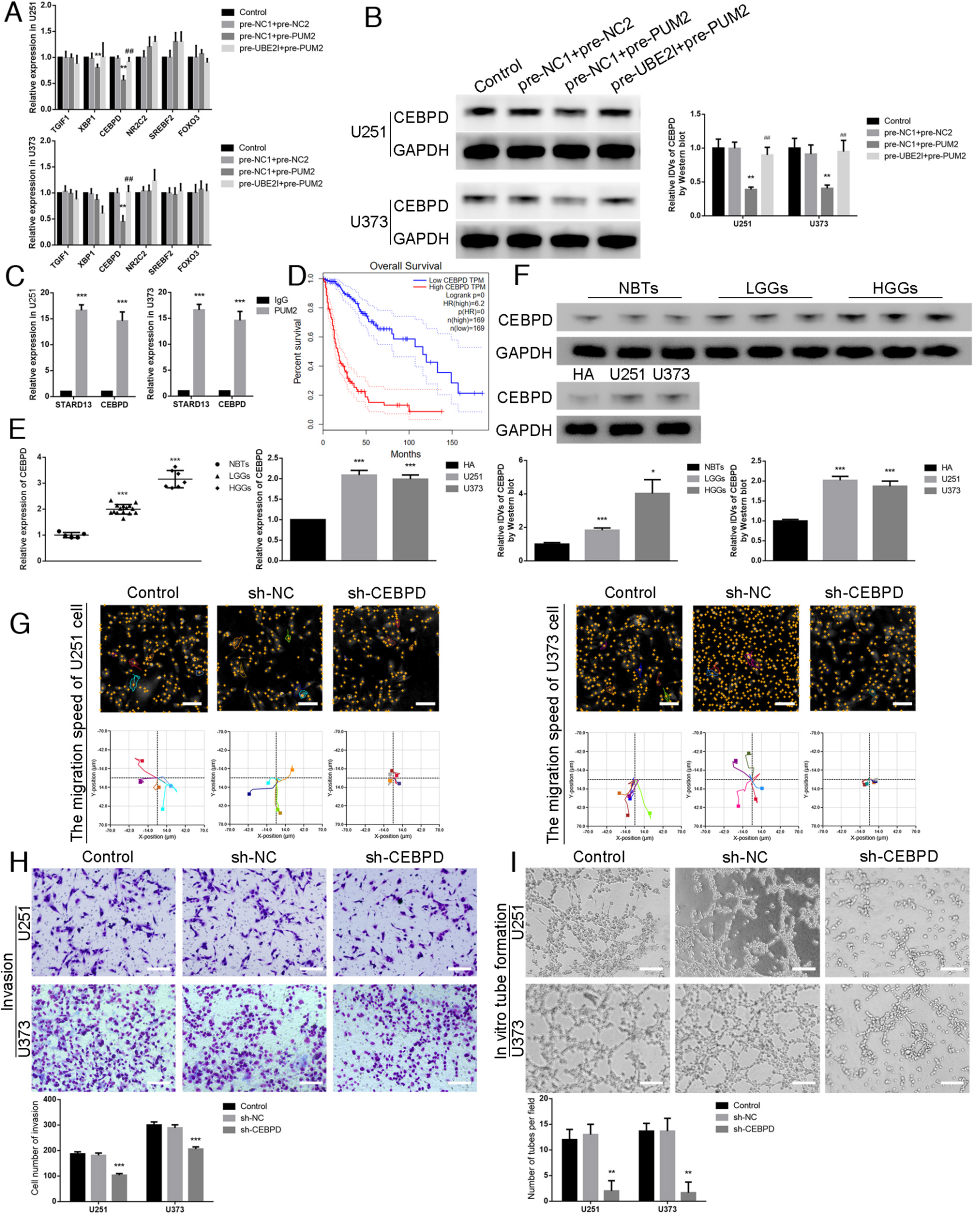
PUM2 inhibited CEBPD expression by binding to CEBPD mRNA, thereby inhibiting the capacities for migration, invasion, and VM in glioma cells:(A) qRT‐PCR was used to detect the changes of TGIF1, XBP1, CEBPD, NR2C2, SREBF2, and FOXO3 mRNA expression in the cells (U251 and U373) overexpressed UBE2I after overexpressed PUM2. Each value represents the mean±SD (n = 3), *P < .05, **P < .01, compared with pre‐NC1+pre‐NC2 group; ##*P <* .01, compared with pre‐NC1+pre‐PUM2 group. (B) Western blot was used to detect CEBPD protein expression in the cells (U251 and U373) overexpressed UBE2I after overexpressed PUM2. Each value represents the mean±SD (n = 3), **P < .01, compared with pre‐NC1+pre‐NC2 group; ##*P <* .01, compared with pre‐NC1+pre‐PUM2 group. (C) RIP assays were used to detect the binding of PUM2 protein to CEBPD mRNA, PUM2 protein to STARD13 mRNA as a positive control, and the IgG group as a negative control. (D) Analysis of the expression of CEBPD in the data of glioma tissue in the TCGA database (n (high) = 169, n (low) = 169), *P <* .001. (E) qRT‐PCR method was used to detect the expression of CEBPD in NBTs, LGGs, and HGGs (left). Each value represents the mean±SD (n = 3), ***P < .001, compared with the NBTs group. The qRT‐PCR detected the expression of CEBPD in HA cells, U251 cells, and U373 cells (right). Each value represents the mean±SD (n = 3), ***P < .001, compared with the HA cells. (F) Western blot was used to detect the expression of CEBPD in NBTs, LGGs, and HGGs (above). Each value represents the mean±SD (n = 3), *P < .05, ***P < .001, compared with NBTs group. Western blot was used to detect the expression of CEBPD in HA cells, U251 cells, and U373 cells (below). Each value represents the mean±SD (n = 3), ***P < .001, compared with HA cells. (G) Hstudio M4 system was used to observe the change of the capacity for migration in cells (U251 and U373) CEBPD knockdown (n = 5). Scale bars: 100 µm. (H) Transwell method was used to detect the change of the capacity for invasion incells (U251 and U373) CEBPD knockdown. Scale bars: 100 µm. (I) Three‐dimensional cell culture method was used to detect the change of VM in the cells (U251 and U373) CEBPD knockdown. Each value represents the mean±SD (n = 3), **P < .01, ***P < .001, compared with sh‐NC group

This study used data from LGG (brain lower grade glioma) and GBM (glioblastoma multiforme) groups in the TCGA database to analyze the genes related to PUM2 (online Additional file 3) and the genes related to UBE2I (online Additional file 4), and to intersect with the transcription factors in the Jaspar database to select the six most relevant transcription factors. Our results showed that UBE2I overexpression rescued CEBPD mRNA and protein expression that was decreased by PUM2 overexpression (Figure 4A,B). Next, the RIP assays were used to detect the binding of PUM2 protein and CEBPD mRNA, the binding of PUM2 protein, and STARD13 mRNA was regarded as a positive control,^25^ and IgG was used as a negative control. The results found that PUM2 can bind to CEBPD mRNA (Figure 4C). By scanning TCGA database, it was found that the expression of CEBPD was negatively correlated with the survival time of the patients (Figure 4D). This study found that the expression of CEBPD in glioma tissue was significantly higher than that in normal brain tissue. The expression in tumor U251 and U373 cells was significantly higher than those in HA cells (Figure 4E,F). Using the Hstudio M4 system, the Transwell method, and the three‐dimensional cell culture method, it was found that CEBPD knockdown significantly inhibited the capacities for migration, invasion, and VM in glioma cells compared with the sh‐NC group (Figure 4G‐I). The results confirmed that the UBE2I/PUM2 axis regulated the expression of CEBPD. Compared with HA cells, CEBPD expression in glioma cells was significantly increased, and CEBPD knockdown significantly inhibited the capacities for migration, invasion, and VM in glioma cells.

### 3.5 CEBPD promoted the capacities for migration, invasion, and VM in glioma cells by regulating the transcription of DSG2

**FIGURE 5:**
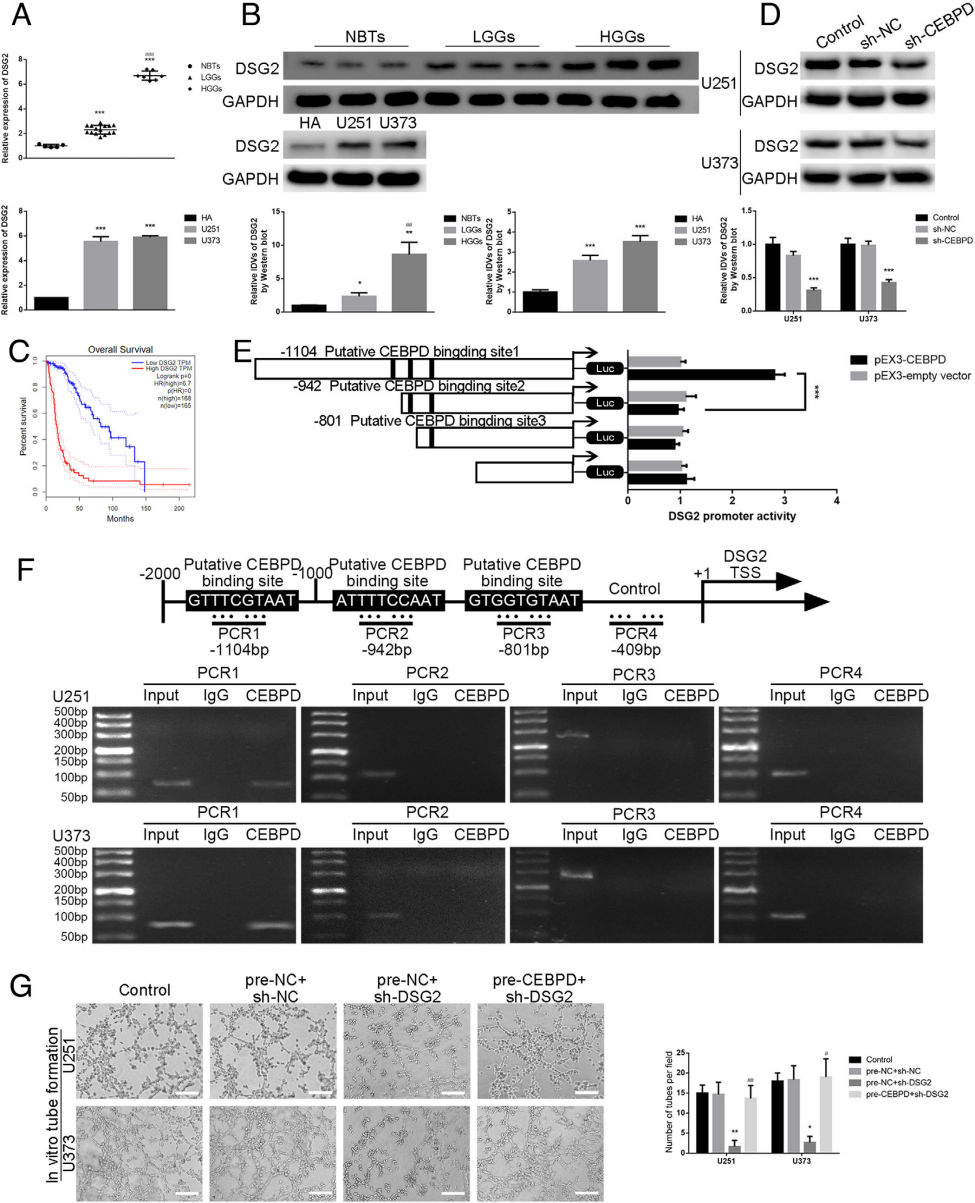
CEBPD promoted the capacities for migration, invasion, and VM in glioma cells through transcriptional regulation of DSG2 expression:(A) qRT‐PCR was used to detect the expression of DSG2 in NBTs, LGGs, and HGGs (above). Each value represents the mean±SD (n = 3), ***P < .001, compared with the NBTs group; ###*P <* .001, compared with the LGGs group. The qRT‐PCR detected the expression of CEBPD in HA cells, U251 cells, and U373 cells (right). Each value represents the mean±SD (n = 3), ***P < .001, compared with the HA cells. (B) Western blot was used to detect the expression of DSG2 in NBTs, LGGs, and HGGs (above). Each value represents the mean±SD (n = 3), *P < .05, **P < .01, compared with the NBTs group; ##*P <* .01, compared with the LGGs group. Western blot method was used to detect the expression of DSG2 in HA cells, U251 cells, and U373 cells (below). Each value represents the mean±SD (n = 3), ***P < .001, compared with the HA cells. (C) Analysis of the expression of DSG2 in the data of glioma tissue in the TCGA database (n (high) = 168, n (low) = 165), *P <* .001. (D) Western blot was used to detect the expression of DSG2 protein in the cells (U251 and U373) CEBPD knockdown. Each value represents the mean±SD (n = 3), ***P < .001, compared with the sh‐NC group. (E) CEBPD affect on DSG2 promoter activity. Each value represents the mean±SD (n = 3), ***P < .001. (F) ChIP experiment detected the binding effect of CEBPD and DSG2 promoter region. (G) Three‐dimensional cell culture method was used to detect the change of VM in the cells (U251 and U373) overexpressing CEBPD after knocking down DSG2. Scale bars: 200 µm. Each value represents the mean±SD (n = 3), *P < .05, **P < .01, compared with the pre‐NC+sh‐NC group; #*P <* .05, ##*P <* .01, compared with the pre‐NC+sh‐DSG2 group

Recently, some scholars have confirmed that the expression of DSG2 in human melanoma is related to the role of tumor VM.^37^ In this study, the expression of DSG2 was significantly increased in the tumor tissue of glioma patients compared with normal brain tissue by qRT‐PCR and western blot, and the expression in glioma U251 and U373 cells was significantly higher than that of HA cells (Figure 5A,B). By scanning TCGA database, it was found that the expression of DSG2 was negatively correlated with the survival time of the patients (Figure 5C). CEBPD knockdown significantly decreased the expression of DSG2 (Figure 5D). As shown in Figure 5E, deletion of the putative binding site 1 (–1104 bp site region) significantly downregulated the promoter activities of DSG2. Further, it was found that there was a CEBPD binding site in the upstream promoter region (–1104 bp site region) of DSG2 gene by ChIP assays (Figure 5F). Through the Hstudio M4 system, Transwell method, three‐dimensional cell culture method, it was found that DSG2 knockdown significantly inhibited the capacities for migration, invasion, and VM in glioma cells compared with the sh‐NC group, and CEBPD overexpression significantly rescued the effect of DSG2 knockdown (online Additional Figure 3A,B and Figure 5G). This study confirmed that CEBPD promoted the expression of DSG2 by transcription in glioma cells, thereby promoting the capacities for migration, invasion, and VM in glioma cells.

### 3.6 UBE2I and CEBPD knockdown combined with PUM2 overexpression inhibited tumor growth, induced the longest survival time in nude mice, and inhibited VM in nude mice

Based on the above results, this study examined the inhibitory effects on migration, invasion, and VM in glioma cells by regulating the expression of UBE2I, PUM2, and CEBPD alone or in combination. Compared with the NC group, UBE2I knockdown, PUM2 overexpression, and CEBPD knockdown significantly inhibited the capacities for migration, invasion, and VM in glioma cells. Moreover, the inhibitory effect on glioma cells when combining the three was higher than the effect of the three alone (online Additional Figure 4).

**FIGURE 6:**
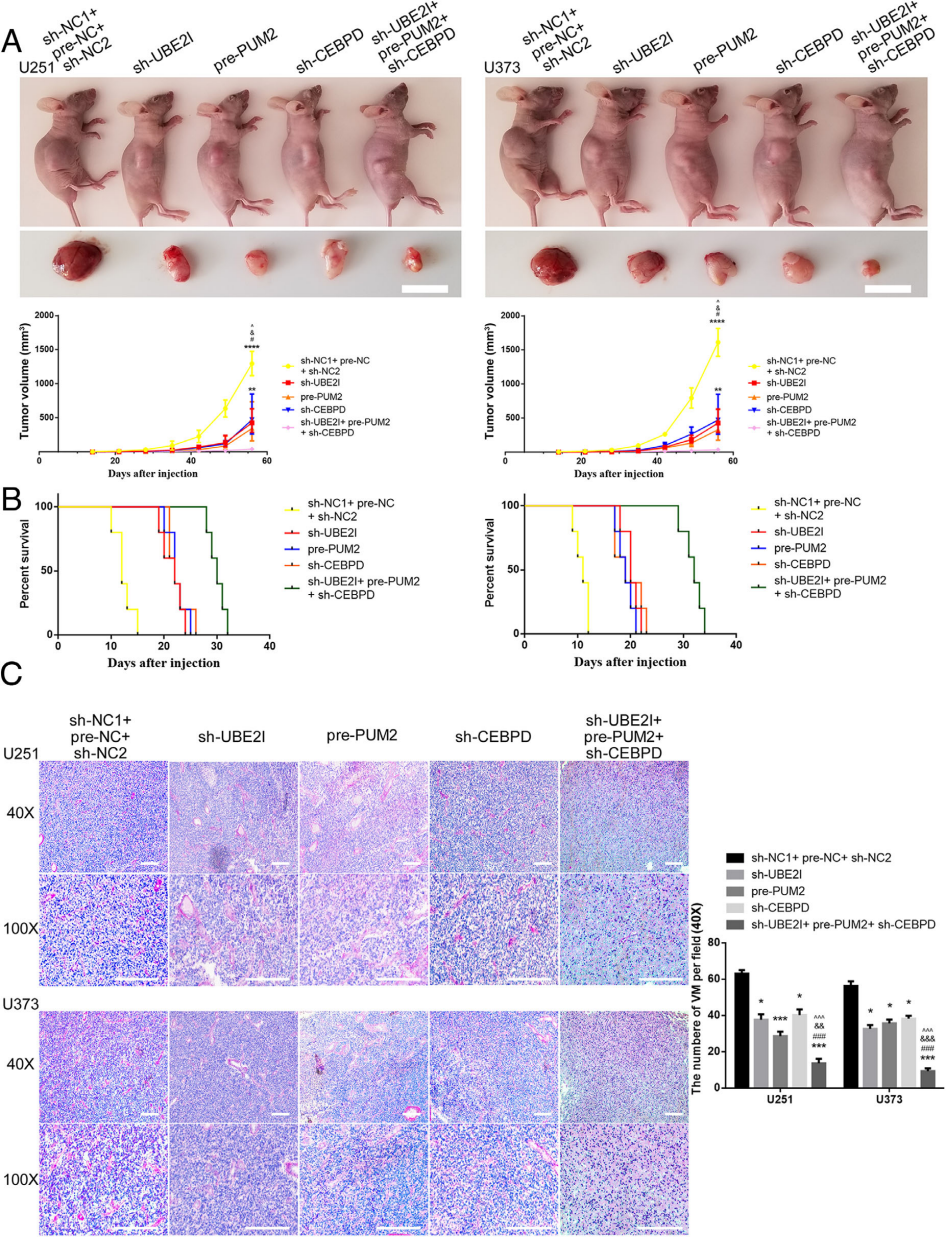
In vivo detection of UBE2I/PUM2/CEBPD axis regulates the capacities for migration, invasion, and VM in glioma cells:(A) Nude mouse subcutaneous transplantation tumor experiment to detect the inhibitory effect of knockdown UBE2I, overexpression PUM2, and knockdown CEBPD on the growth volume of glioma alone and in combination. Scale bars: 1 cm. Each value represents the mean±SD (n = 3), **P < .01, ****P < .0001, compared with the sh‐NC1+pre‐NC+sh‐NC2group; #*P <* .05, compared with the sh‐UBE2I group; &*P <* .05, compared with the pre‐PUM2 group; ^*P <* .05, compared with the sh‐CEBPD group. (B) The orthotopic xenograft experiment in nude mice detected the effects of UBE2I knockdown, PUM2 overexpression, and CEBPD knockdown, alone or in combination, on the survival time of nude mice. (C) CD34‐PAS staining was used to detect the effects of UBE2I knockdown, PUM2 overexpression, and CEBPD knockdown on VM in orthotopic transplanted tumor tissue of nude mice, alone or in combination. Scale bars: 200 µm. Each value represents the mean±SD (n = 3), *P < .05, ***P < .001, compared with the sh‐NC1+pre‐NC+sh‐NC2 group;###*P <* .001, compared with the sh‐UBE2I group; &&*P <* .01, &&&*P <* .001, compared with the pre‐PUM2group; ^^^*P <* .001, compared with the sh‐CEBPD group

Finally, in this study, the nude mice transplantation experiment was used to detect the role of the UBE2I/PUM2/CEBPD axis in the antitumor function. In nude mice with subcutaneous xenografts, the volume of subcutaneous xenografts in nude mice with UBE2I knockdown, PUM2 overexpression, and CEBPD knockdown decreased significantly compared with the NC group (Figure 6A,B). Additionally, PUM2 overexpression combined with knockdown of both UBE2I and CEBPD resulted in the smallest tumor volume among all the groups. The Kaplan‐Meier method was further used to record the survival time of each group of orthotopic mice brain xenografts. Compared with the NC group, the nude mice survival time of UBE2I knockdown, PUM2 overexpression, and CEBPD knockdown was significantly prolonged, and the time of the three coregulation was significantly longer than among all the groups (Figure 6C). Finally, in this study, pathological sections of orthotopically transplanted nude mice were taken. Further, CD34‐PAS staining found that the number of VMs in sh‐UBE2I group, pre‐PUM2 group, sh‐CEBPD group, and sh‐UBE2I+ pre‐PUM2 +sh‐CEBPD group was decreased compared with NC group. Meanwhile, sh‐UBE2I+ pre‐PUM2 +sh‐CEBPD group was significantly decreased compared with sh‐UBE2I group, pre‐PUM2 group, and sh‐CEBPD group, respectively.

## 4 DISCUSSION

**FIGURE 7:**
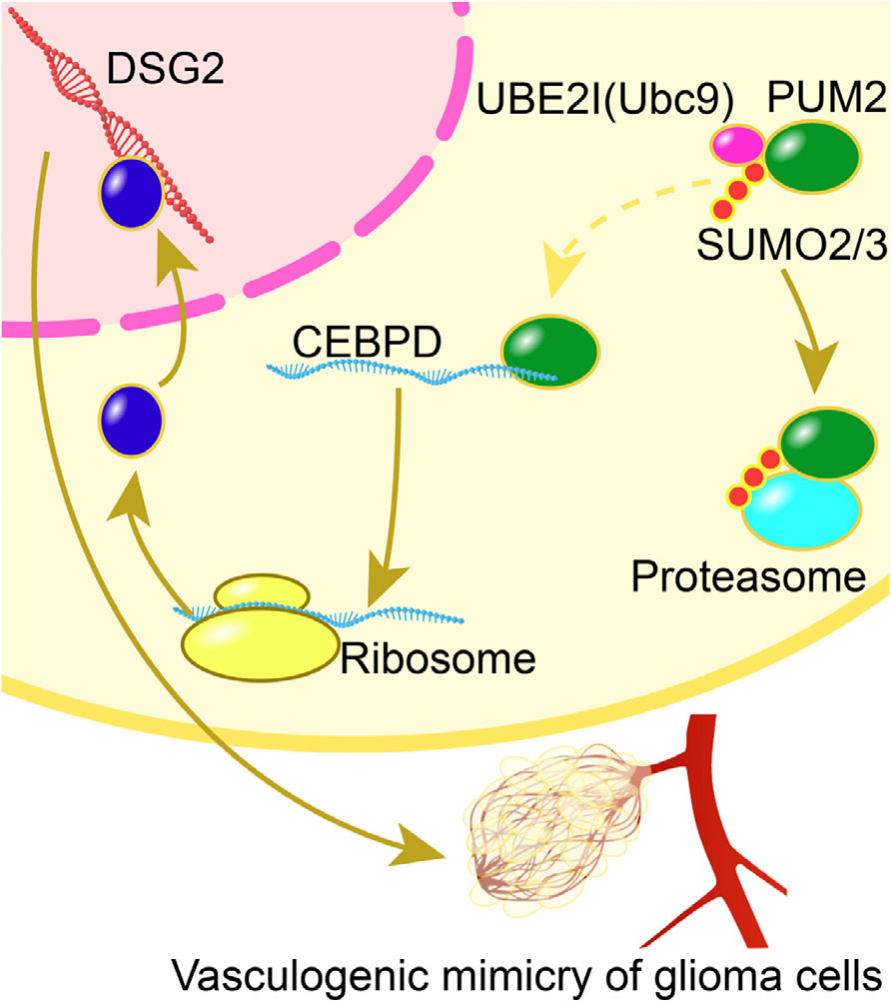
The expression of UBE2I in glioma cells is significantly increased, thereby promoting PUM2 SUMOylation, leading to the degradation of PUM2 protein by proteasome. UBE2I inhibits the role of PUM2 protein in the degradation of CEBPD mRNA. CEBPD overexpression promotes the transcriptional expression of DSG2, which in turn promotes the capacities for migration, invasion, and VM in glioma cells

This study found that the expression of UBE2I was significantly increased in glioma cells, which promoted PUM2 SUMOylation, led to the degradation of PUM2 protein, and inhibited the role of PUM2 protein in the degradation of CEBPD mRNA. CEBPD overexpression promotes the transcriptional expression of DSG2, which in turn promotes the capacities for migration, invasion, and VM in glioma cells (Figure 7).

The Ubc9 protein translated from UBE2I gene is the only SUMO‐conjugating enzyme and participates in the process of SUMOylation. SUMOylation regulates protein function by covalently binding to lysine residues in the target protein, which is similar to ubiquitination.^15^, ^39^ It was previously thought that SUMOylation is involved in the regulation of various intracellular protein biological functions, such as regulating the activity of transcription factors,^40^ controlling protein phase separation,^41^ mediating the nuclear translocation of proteins or the formation of subnuclear structures,^42^, ^43^ and increasing protein stability.^44^ However, in the study of APL, it is found that arsenic‐induced PML SUMOylation triggers polyubiquitination at Lys^48^ and proteasome‐dependent degradation.^45^ During heat shock response, the stress‐induced SUMO2/3 target protein can be degraded by the ubiquitin‐proteasome system, thereby coordinating proteomic degradation and helping maintain protein homeostasis during protein toxicity stress.^46^ In this study, we found that PUM2 protein and SUMO2/3 protein were colocalized in glioma cells by laser scanning confocal microscopy. Furthermore, through GPS‐SUMO (http://hemi.biocuckoo.org) website prediction and Co‐IP, it was found that PUM2 protein can be modified by SUMO2/3 protein at position K^50/91/985^ to undergo SUMOylation, and be degraded. By scanning TCGA database, we found that the expression of UBE2I in the data of glioma patients was negatively correlated with the survival time of patients. At the same time, we found that UBE2I expression was increased in glioma tissues and cells, and was positively correlated with glioma grade. UBE2I knockdown significantly promoted the expression of PUM2 protein in the cytoplasm, but did not significantly affect the nuclear and cytoplasmic distribution of PUM2 protein, while inhibiting the capacities for migration, invasion, and VM in glioma cells. These results detected that UBE2I exerts a cancer‐promoting effect by PUM2 SUMOylation in glioma cells.

As a member of the PUF protein family, PUM2 participates in maintaining genomic integrity^47^ and regulating RNA stability,^22^ thereby regulating the occurrence and development of various physiological and pathological processes. Recent studies have shown that PUM2 in mice inhibits translation by binding to PBE in the 3′UTR of Cdkn1b, promotes G1‐S transition, promotes cell proliferation, and thereby regulates the body and organ size of mice.^48^ In cervical cancer, lncRNA TUG1 promotes the upregulation of PUM2 expression through interaction with PUM2, enhancing the proliferation and migration of cervical cancer cells.^49^ In breast cancer, PUM2 promotes the stemness of breast cancer cells by competitively binding NRP‐1 3′UTR with miR‐376a.^24^ In osteosarcoma, the expression of PUM2 is significantly decreased. Overexpression of PUM2 can inhibit the tumor progression by competitively binding to the 3′UTR of STARD13 with miR‐590‐3p and miR‐9^25^. In this study, by scanning TCGA database, it was found that the expression of PUM2 was positively correlated with the survival time of the patient, and found that PUM2 expression was decreased in glioma tissues. Further, it was found that the expression of PUM2 was significantly decreased in the cytoplasm of glioma. Overexpression of PUM2 inhibited the capacities for migration, invasion, and VM in glioma cells, and our experimental results found that UBE2I inhibited the expression of PUM2 protein through SUMOylation, further promoting the capacities for migration, invasion, and VM in glioma cells.

In this study, by analyzing the database and experimental results, it was found that PUM2 can regulate the VM of glioma cells by binding CEBPD mRNA. Studies have shown that CEBPD can regulate the biological behavior of many cells, including cell differentiation, migration, invasion, growth arrest, proliferation, and apoptosis.^50^ CEBPD promotes the proliferation of breast cancer stem cells by activating IL‐6 and HIF‐1 in breast cancer.^51^ Extensive fibrosis reaction around pancreatic cancer and within the tumor is positively correlated with CEBPD expression in pancreatic cancer specimens.^52^ CEBPD is a vital transcriptional regulator in ovarian cancer. It promotes cell migration, EMT/MET, and cell survival by the IL‐6/STAT3 signaling pathway, while maintaining genome stability.^53^ In gliomas, DN‐ATF5 acts as a tumor suppressor by blocking the transcriptional activity of CEBPD.^54^ CEBPD can also maintain the stemness of glioma stem cells by promoting PDGFA expression.^55^ This study shows that CEBPD is highly expressed in glioma tissues and cells, and the TCGA database search revealed that CEBPD expression was negatively correlated with patient survival. Further results showed that the UBE2I/PUM2 axis regulates the expression of CEBPD protein. In addition, CEBPD knockdown inhibited the capacities for migration, invasion, and VM in glioma cells, and detected that it plays a procancer role in gliomas.

DSG2, as a desmosomal cadherin, is abnormally expressed in various tumor tissues, and is mainly involved in regulating the proliferation, metastasis, tumor microenvironment, and tumor VM of tumor cells. Analysis of human colon cancer shows that DSG2 protein expression is increased and mediates the proliferation of colon cancer cells through the EGFR signaling pathway.^56^ DSG2 pretreated breast cancer cells have decreased cell aggregation, increased invasion, and motility in vitro.^57^ Furthermore, DSG2 regulates the release of extracellular vesicles of squamous cell carcinoma keratinocytes and regulates the tumor microenvironment by this mechanism.^35^ Moreover, overexpression of DSG2 in lung adenocarcinoma and squamous cell carcinoma cells activates EGFR and increases cancer cell proliferation and migration by c‐Src and EGFR‐dependent manner.^58^, ^59^ Recent research confirms that the expression of DSG2 in human melanoma is positively correlated with tumor VM^37^F. In this study, DSG2 is overexpressed in glioma tissues and cells, and the expression is negatively related to the survival time of patients. CEBPD knockdown significantly decreased the expression of DSG2 protein. At the same time, through the ChIP assays, it was found that CEBPD specifically bounds to the DSG2 gene promoter region, thereby transcriptionally regulating the expression of DSG2 protein. And we found that the CEBPD/DSG2 axis regulates the capacities for migration, invasion, and VM in glioma cells. However, the mechanism by which DSG2 affects the capacity for VM needs further study.

This study found that the expression of UBE2I, CEBPD, and DSG2 in glioma tissues and cells was significantly increased, and was significantly negatively correlated with the survival prognosis of glioma patients, while the expression of PUM2 was significantly decreased, and was significantly positively correlated with the survival of glioma patients. Laser scanning confocal microscopy and Co‐IP assays confirmed that UBE2I bound to PUM2, promoted PUM2 SUMOylation, and then regulated the expression of PUM2 protein. Database analyzing, qRT‐PCR, and RIP assays were used to confirm that PUM2 regulated the expression of CEBPD protein by binding CEBPD mRNA. ChIP assays were used to confirm that CEBPD promoted the expression of DSG2 by binding transcription with DSG2 gene promoter region. This study found that the knockdown of UBE2I, CEBPD, and DSG2, and the overexpression of PUM2 in vitro all inhibited the capacities for migration, invasion, and VM in glioma cells. Further, nude mice transplantation experiment in vivo found that the knockdown of UBE2I, CEBPD, and overexpressed PUM2 alone inhibited the growth of transplanted tumors, prolonged the survival time of nude mice, and inhibited the VM in transplanted tumors, and the effect of three coregulation was the most significant.

In summary, this study first discovered the abnormal expression of UBE2I, CEBPD, PUM2, and DSG2 in glioma tissues and cells. UBE2I knockdown, CEBPD knockdown, and PUM2 overexpression significantly inhibited the capacities for migration, invasion, and VM in glioma cells. UBE2I knockdown inhibited PUM2 SUMOylation, resulting in increased stability of PUM2 protein and increased expression of PUM2 protein in the cytoplasm. Further, PUM2 overexpression bound to the PRE of CEBPD mRNA and promoted the degradation of CEBPD. CEBPD knockdown inhibited the transcriptional regulation of the DSG2, thereby inhibiting the capacities for migration, invasion, and VM in glioma cells. The results of this study confirmed that UBE2I, CEBPD, and PUM2 have the potential to be new molecular targets, and the research on the treatment of gliomas needs to be further explored.

## Supporting information

SUPPORTING INFORMATION

SUPPORTING INFORMATION

SUPPORTING INFORMATION

SUPPORTING INFORMATION

SUPPORTING INFORMATION

SUPPORTING INFORMATION
