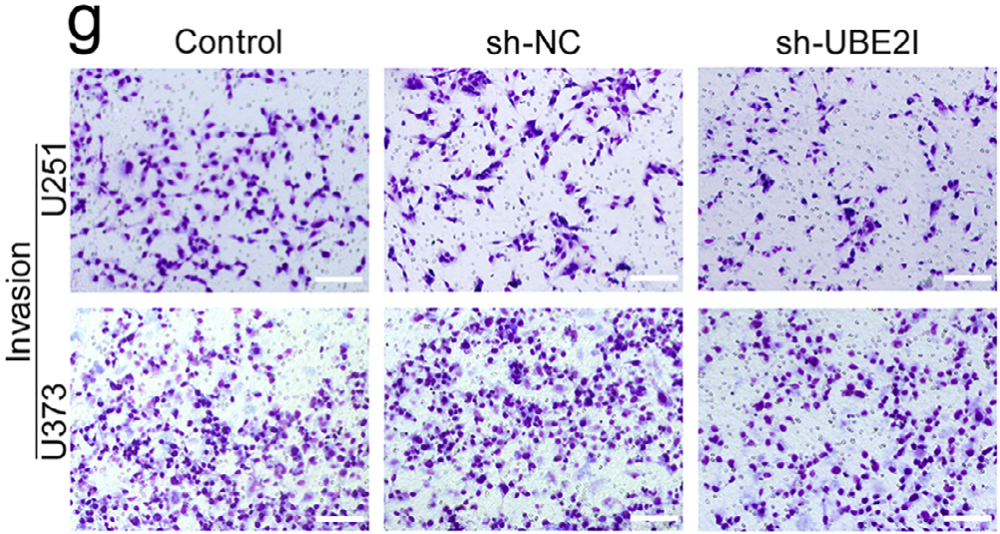# Correction to ‘SUMOylation of PUM2 promotes the vasculogenic mimicry of glioma cells via regulating CEBPD’

**DOI:** 10.1002/ctm2.70559

**Published:** 2026-01-02

Wang D, Ruan X, Liu X, et al. SUMOylation of PUM2 promotes the vasculogenic mimicry of glioma cells via regulating CEBPD. *Clin Transl Med*. 2020;10(5):e168. doi:10.1002/ctm2.168

**[Description of error]**

In Figure 2g, the image of the U251 control group was inadvertently taken from an earlier draft version rather than the finalised version. Consequently, the control group image may appear to display a slightly higher apparent cell density compared with the sh‐NC group, which could lead to a minor visual misinterpretation.

After carefully re‐examining all original data, we confirmed that this mistake was due to the incorrect selection of a representative image for the control group. Therefore, this correction does not alter the results, statistical analyses, or conclusions of the paper.

The corrected version of Figure 2g is provided below. We sincerely apologise for this oversight and for any confusion it may have caused.