# Supplementary material for: SUMOylation of PUM2 promotes the vasculogenic mimicry of glioma cells via regulating CEBPD
Source: Clin Transl Med. 2020 Sep 6;10(5):e168. doi: 10.1002/ctm2.168 (PMC7507322; doi:10.1002/ctm2.168)
Supplement: Supplementary file 1 — SUPPORTING INFORMATION [file CTM2-10-e168-s001.pdf]

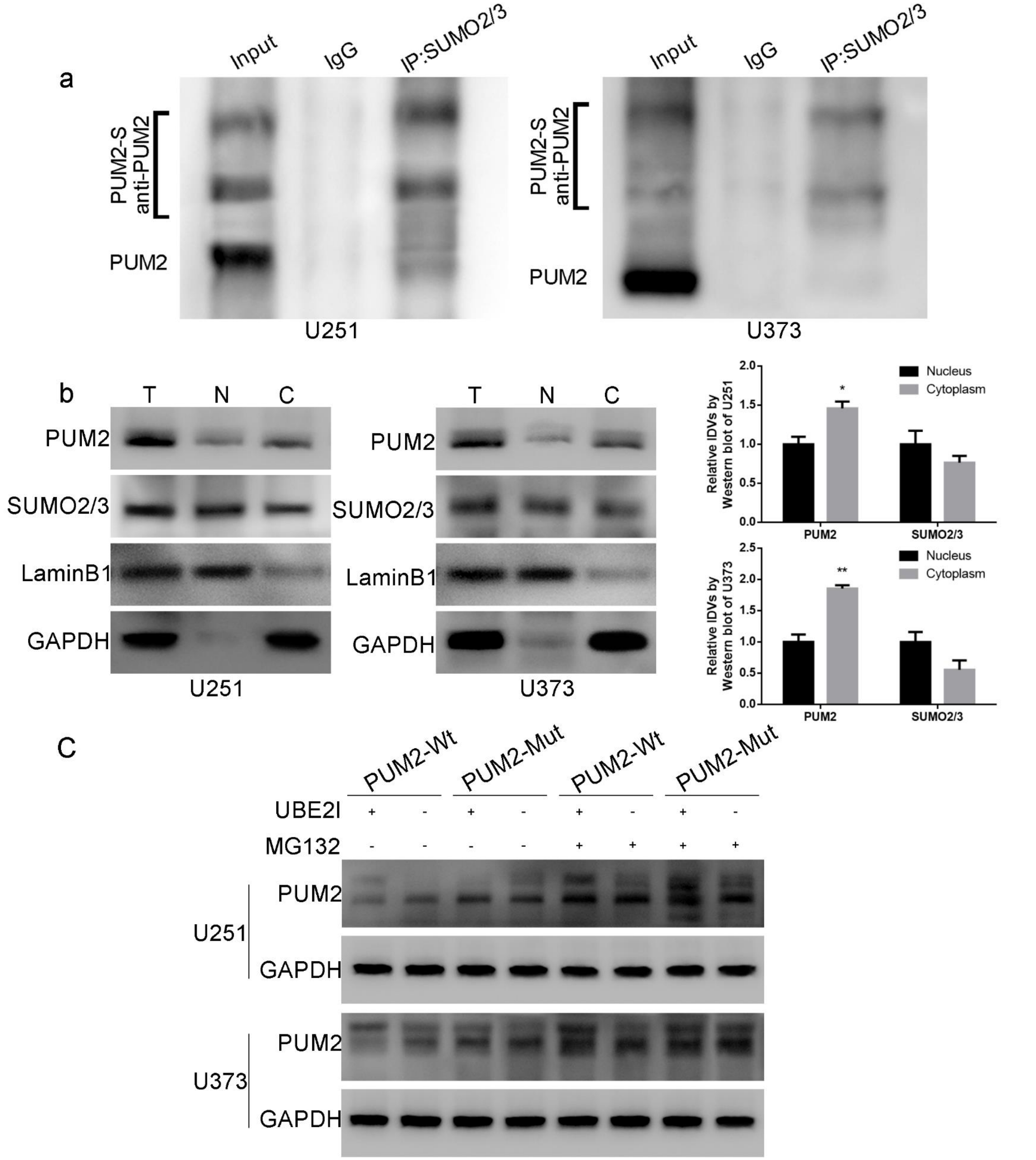

Additional Figure 1. (a) PUM2 was modified by SUMO2/3 in U251 cells and U373 cells. The Co-IP experiment detected that SUMO2/3 protein bound to the PUM2 protein, and followed by western blot with indicated antibodies.(b) Western blot was used to evaluate the subcellular localization of PUM2 and SUMO2/3 in U251 and U373 cells. T, Total protein; N, Nuclear protein; C, Cytoplasmic protein. Each value represents the mean±SD (n=3), *P<0.05, **P<0.01, compared with the Nucleus group. (c) Western blot was used to evaluate that MG132 inhibits the degradation of PUM2 by SUMOylation.

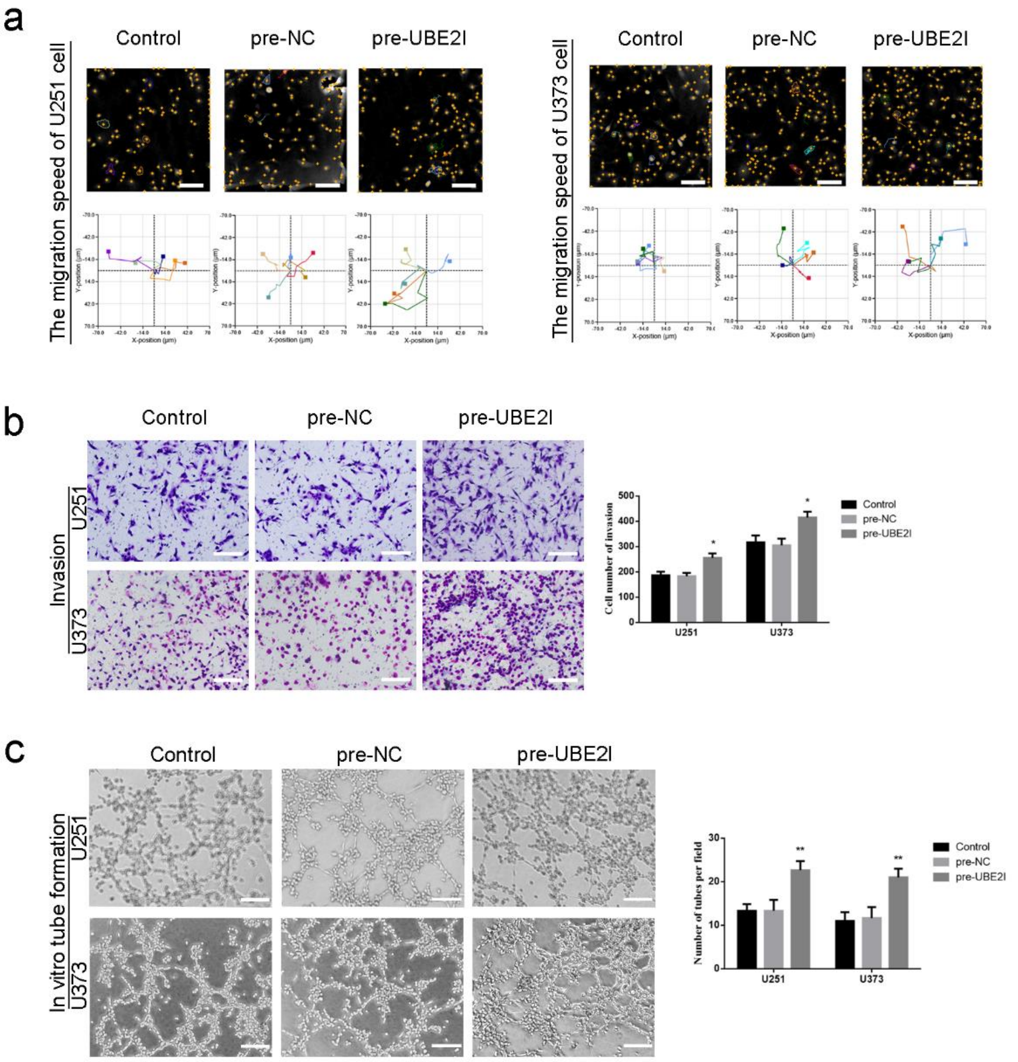

Additional Figure 2. (a) The Hstudio M4 system observed the migration ability of the UBE2I overexpressed cells (U251 and U373), (n=5). Scale bars: 100μm. (g) Transwell method was used to detect the invasion ability of the UBE2I overexpressed cells (U251 and U373). Scale bars: 100μm. (h) Three-dimensional cell culture method was used to detect the change of VM in the UBE2I overexpressed cells (U251 and U373). Scale bars: 200μm. Each value represents the mean±SD (n=3), *P<0.05, **P<0.01, compared with pre-NC group.

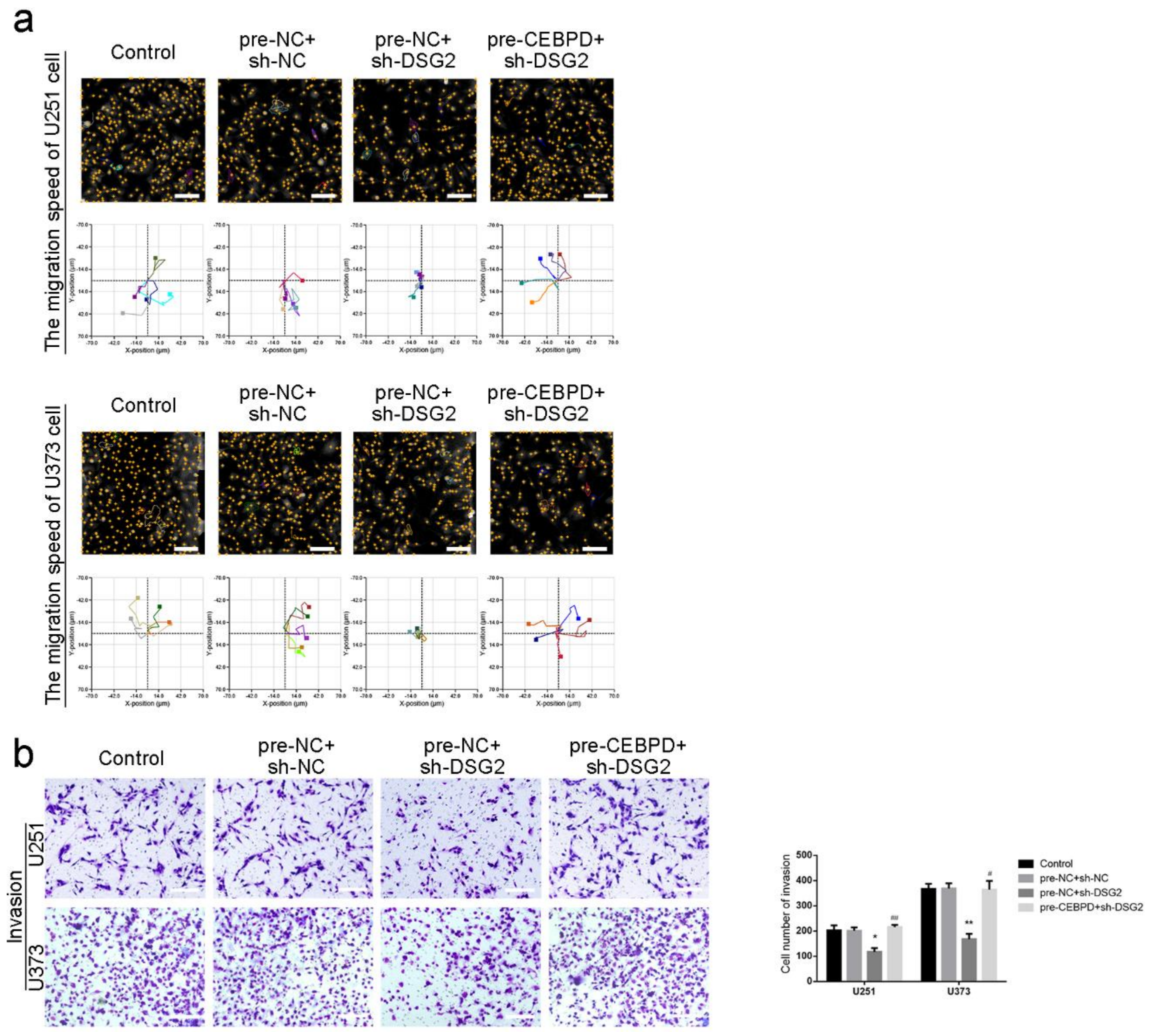

Additional Figure 3. (a) Hstudio M4 system was used to observe the change of migration ability of U251 cells and U373 cells overexpressing CEBPD after knocking down DSG2 (n=5). bar=100μm. (b) Transwell method was used to detect the invasion ability of U251 cells and U373 cells overexpressing CEBPD after knocking down DSG2. bar = 100μm. Each value represents the mean±SD (n=3), **P*<0.05, ***P*<0.01, compared with the pre-NC+sh-NC group; #*P*<0.05, ##*P*<0.01, compared with the pre-NC+sh-DSG2 group.

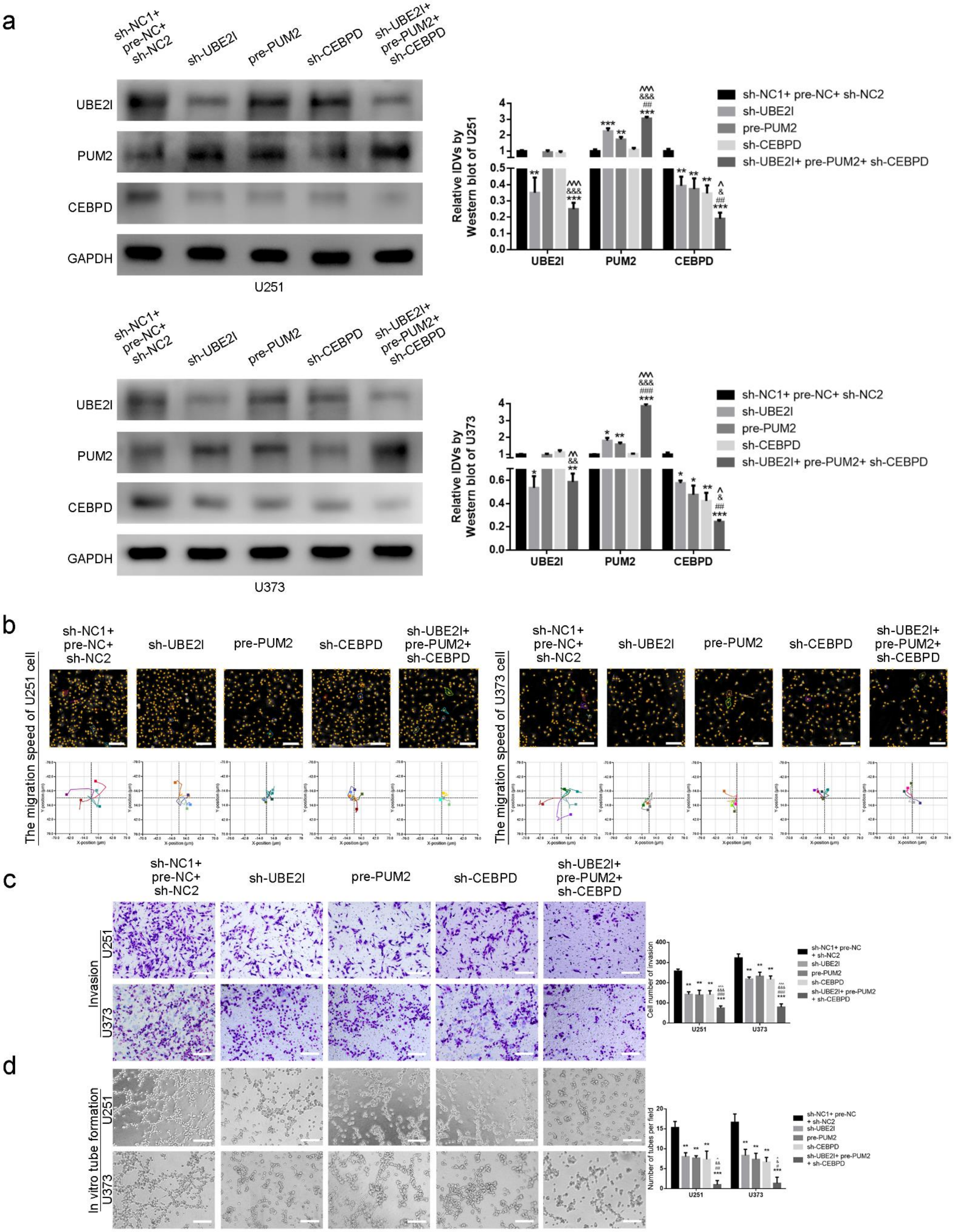

Additional Figure 4. (a) Western blot was used to evaluate the protein expression of UBE2I, PUM2, and CEBPD in

U251 cells and U373 cells used for transplantation tumor experiments. (b) The Hstudio M4 system was used to observe the changes in migration ability of U251 cells and U373 cells (n=5) for UBE2I knockdown, PUM2 overexpression, and CEBPD knockdown alone and in combination. bar= 100μm. (c) Transwell method was used to detect the changes of U251 cells and U373 cells invasion ability of UBE2I knockdown, PUM2 overexpression and CEBPD knockdown alone and in combination. bar=100μm. (d) Three-dimensional cell culture method was used to detect the change of VM in U251 cells and U373 cells applied alone and in combination with UBE2I knockdown, PUM2 overexpressed and CEBPD knockdown. bar=200μm. Each value represents the mean±SD (n=3), *$P<0.05$, **$P<0.01$, ***$P<0.001$, compared with the sh-NC1+pre-NC+sh-NC2 group; ##$P<0.01$, ###$P<0.001$, compared with the sh-UBE2I group; &$P<0.05$, &&$P<0.01$, &&&P<0.001, compared with the pre-PUM2 group; ^$P<0.05$, ^^$P<0.01$, ^^^$P<0.001$, compared with the sh-CEBPD group.
