# Supplementary material for: SUMOylation of PUM2 promotes the vasculogenic mimicry of glioma cells via regulating CEBPD
Source: Clin Transl Med. 2020 Sep 6;10(5):e168. doi: 10.1002/ctm2.168 (PMC7507322; doi:10.1002/ctm2.168)
Supplement: Supplementary file 6 — SUPPORTING INFORMATION [file CTM2-10-e168-s006.pdf]

Additional Materials and Methods

Pathological tissue acquisition and cell culture

Human glioma tissues and normal brain tissues were collected from patients at the Department of Neurosurgery of Shengjing Hospital of China Medical University. All the tissue samples were immediately frozen in liquid nitrogen after surgical resection and stored at -80°C until use. All patients voluntarily signed informed consent, and the study was approved by the Ethics Committee of Shengjing Hospital of China Medical University. Glioma tissue samples were divided into two groups: low-grade glioma tissues and high-grade glioma tissues by neuropathologists according to the 2007 WHO classification of tumors in the central nervous system. Human astrocyte (HA) cells were obtained from the Shanghai Zeye Biotechnology and grown in RPMI-1640 culture medium (Gibco, Grand Island, NY, USA) with 10% fetal bovine serum (FBS, Gibco, Carlsbad, CA, USA). Human glioma cells (U251 and U373) and human embryonic kidney (HEK) 293T cells were purchased from Shanghai Genechem Co, Ltd, and grown in Dulbecco's modified Eagle medium (DMEM)/high glucose with 10% FBS. All cells were maintained in a humidified incubator at 37°C with 5% CO_2.

Quantitative real-time PCR (qRT PCR)

Total RNA was extracted from tissues and cells (U251 and U373) with Trizol reagent (Life Technologies Corporation, Carlsbad, CA, USA). RNA concentration and

quality were determined via 260/280 nm absorbance with Nanodrop Spectrophotometer (ND-100, Thermo, USA). Using 7500 Fast RTPCR System, One-Step SYBR Prime-Script RT-PCR Kit (TakaraBio, Inc., Japan) was used to detect the expression of UBE2I (NM_003345.5), PUM2 (NM_001282752.2), CEBPD (NM_005195.4) and DSG2 (NM_001943.5), meanwhile, GAPDH was used as the endogenous control. See Additional file 1 for details and primers used.

Cell transfection

Short hairpin RNA (shRNA) directed against human UBE2I, PUM2, CEBPD and DSG2 gene were constructed in pGPU6/GFP/Neo vector (Gene-Pharama, Shanghai, China). The full-length UBE2I, PUM2 (and PUM2-Mut ($K^{50/91/985}R$, 3KR)) and CEBPD gene were constructed in pIRES2-EGFP (JTS, Beijing, China). Plasmid carrying a non-targeting sequence was used as a negative control. When cells reached 70–80% confluence, the transaction was performed by Lipofectamine 3000 Reagents (Life Technologies, Carlsbad, CA, USA). G418, hygromycin and puromcin (Sigma-Aldrich, StLouis, MO, USA) were used to select the stable transfected cells. Resistant cell clones were established around approximately 4 weeks. Transfected efficiencies of stable cell lines were analyzed by qRT-PCR.

RNA binding protein immunoprecipitation assays (RIP)

Based on the manufacturer’s protocols, a RIP assays was carried using an EZ-Magna RNA-binding protein immunoprecipitation kit (Millipore, USA) in this

study. The whole-cell lysate was incubated with RIP buffer containing magnetic beads conjugated with human anti-PUM2 antibody (Abcam, UK), PUM2 protein to STARD13 mRNA as a positive control, and the IgG group as a negative control. Samples were incubated with Proteinase K and immunoprecipitated RNA was isolated. The RNA concentration was measured by a spectrophotometer (NanoDrop, Thermo Scientific, Waltham, MA, USA) and the RNA quality was assessed using a bioanalyzer (Agilent, Santa Clara, CA, USA). Furthermore, purified RNAs were extracted and analyzed by qRT-PCR to demonstrate the presence of the binding targets.

Western blot

Harvested Cells (U251 and U373) were lysed using RIPA (Beyotime Institute of Biotechnology) buffer on ice for 30 min and were centrifuged at 17,000×g for 45 min at 4°C. Nucleo-cytoplasmic separation assays used Nuclear and Cytoplasmic Protein Extraction Kit (Beyotime Institute of Biotechnology) to separate nuclei and cytoplasmic proteins. The protein concentrations were measured by the BCA protein assays kit (Beyotime Institute of Biotechnology, Jiangsu, China). The proteins were processed by SDS-PAGE electrophoretically transferring to PVDF membranes. The membranes were blocked by Tween-Tris-buffered saline (TTBS) containing 5% non-fat milk for 2h at room temperature and then incubated with primary antibodies as follows: SUMO1 (Santa Cruz Biotechnology), SUMO2/3 (Santa Cruz Biotechnology), UBE2I (Proteintech, USA), PUM2 (Abcam, UK), CEBPD

(Proteintech, USA), DSG2 (Proteintech, USA) and GAPDH (Proteintech, USA) overnight at 4°C. After washing three times with TTBS, membranes were incubated with horseradish peroxidase conjugated secondary antibody for 2h at room temperature and then developed with enhanced chemiluminescence (ECL) kit (Santa Cruz Biotechnology) and scanned by ChemImager 5500 V2.03 software according to the manufacturer's protocol. The relative integrated density values (IDVs) were calculated using Fluor Chen 2.0 software based on GAPDH as an internal control.

Co-Immunoprecipitation (Co-IP)

Endogenous SUMOylated PUM2 were detected by immunoprecipitations. Briefly, harvested cells were using RIPA (Beyotime Institute of Biotechnology) buffer on ice for 60 min and incubated with the antibody-coupled beads overnight at 4°C. Beads were washed three times with high-salt buffer. Finally, the beads were boiled for 10 min in SDS sample buffer, and followed by western blot.

Immunofluorescence staining

The cells grown on the surface of coverslips were fixed with 4% paraformaldehyde under the room temperature, followed by permeabilization with 0.2% Triton X-100, and then blocked with 10% goat serum in PBS. Next, coverslips were incubated with primary antibody diluted in 5% goat serum in PBS at 4°C overnight. The cells were washed three times with PBS and then incubated with fluorescent dye-conjugated secondary antibody diluted in 5% goat serum in PBS for

2h away from light. Furthermore, cells were washed three times with PBS and then stained with DAPI for 1h. The immunofluorescence images were recorded by a laser scanning confocal microscopy.

Cell migration assays

Cell migration assays was performed using the HoloMonitor M4 culture system (Phase Holographic Imaging PHI AB, SE) according to the manufacturer's protocols. The cells of each group were inoculated into a six-well plate at a concentration of 2×10^4 cells/ml. After the cells were attached to the petri dish, they were placed on the HoloMonitor M4 culture system and set for imaging for 6h at 1h intervals. For each experimental group, we show the last image frame and the cell movements. At the start of the analysis 5 visually identifiable cells in each experimental set were selected for tracking. Their movements are displayed in spatial X-Y plots.

Cell invasion assays

The cells invasion were assessed by penetrating 8-μm pore size polycarbonate membrane (Costar, Corning, NY, USA). The cells were resuspended in serum-free medium at a density of 2×10^5 cells/ml and seeded into the upper chamber of polycarbonate membrane (pre-coated with Matrigel and incubated at 37°C for 30 min before the invasion assays started). Then, 600 μl of 10% FBS medium was placed into the lower chamber. After incubation at 37°C for 36h, the cells invaded from the upper chamber to the lower surface of the membrane and the cells were fixed with methanol

and glacial acetic acid at a ratio of 3:1, before staining with 20% Giemsa. Five random fields were chosen to count and take photos under a microscope.

Cells VM formation assays

Each hole in the 96-well culture plate was covered with 100μl Matrigel Basement Membrane Matrix (BD Biosciences, Bedford, MA, USA). Meanwhile, the bubbles were avoided. The 96-well culture plate was incubated for 30 min in the incubator at 37℃. The cells were resuspended in 100μl of serum-free medium and seeded onto the surface of Matrigel at a density 6×10^5 cells/ml and incubated for 8 hours. The cells vascular structures were observed and photographed under an inverted microscope (Olympus, Tokyo, Japan). An independent observer counted the total number of tube-like structures per image.

Luciferase assays

The responsive CEBPD-binding sites in the DSG2 promotor were predicted by bioinformatics tool JASPAR and were determined by dual-luciferase reporter system. Promoter fragments were subcloned into pGL3-Basic-Luciferase vector (Promega, WI, USA). Human full-length DSG2 was constructed in pEX3 vector (GenePharma, Shanghai, China). The assays was performed 48h after transfection the indicated constructs into 2.4×10^4 293T cells per well seeded into 96-well plates. The cells were analyzed by the luciferase assays using the dual-luciferase reporter assays system. The relative luciferase activity was expressed as the ratio of firefly luciferase activity to

renilla luciferase activity.

Chromatin immunoprecipitation assays (ChIP)

ChIP assays were performed using the Simple ChIP Enzymatic Chromatin IP Kit (Cell Signaling Technology, Danvers, MA, USA) according to the manufacturer's protocols. Briefly, cells were crosslinked with EBM-2 containing 1% formaldehyde and collected in a lysis buffer containing 1% PMSF. Chromatin was digested by micrococcal nuclease, and 2% aliquots of lysate were used as an input control. Lysates were incubated with 3 μg anti- CEBPD antibody (Proteintech) or normal rabbit IgG, followed by immunoprecipitation with protein G agarose beads and incubation at 4 °C overnight with gentle shaking. DNA crosslink was reversed by the addition of 5 mol/L NaCl and Proteinase K at 65 °C for 2 h, and finally, DNA was purified. Immunoprecipitated DNA was amplified by qRT-PCR using specific primers. See Additional file 2 for details and primers used.

CD34 endothelial marker periodic acid-Schiff dual staining (CD34-PAS)

CD34-PAS was examined for the existence of VM. Human glioma tissue samples were fixed with 4% formaldehyde, embedded in paraffin and sectioned into 5μm tumor slides. These were deparaffinized in xylene, hydrated, and boiled in EDTA antigen-unmasking solution. When cooled to room temperature, slides were incubated in peroxide at room temperature for endogenous peroxidase ablation, blocked with goat serum, and stained with a rabbit anti-human CD34 primary monoclonal antibody

(Beijing Zhongshan Golden bridge, China) overnight at 4℃. After washing with PBS thrice and incubating with goat-anti-rabbit secondary antibody at room temperature for 10 min, the slides were treated with a DAB kit (Fuzhou MaiXin Biotech, China). Then, the slides were exposed to periodic acid solution for 10 min, incubated with Schiff solution for 10 min in the dark, and counterstained with Mayer's hematoxylin (Zhuhai Baso, Guangdong, China). Lastly, the slides were viewed under a light microscope to detect CD34 and PAS signals and were counted the pipe structure of CD34 (-) + PAS (+). Immunohistochemical staining was performed with the help of UltraSensitive S-P kit (Fuzhou MaiXin Biotech, China).

Tumor xenograft implantation in nude mice

For the in vivo study, the stably transfected cells (U251 and U373) were used. The mice were divided into five groups. After infection, the stable expressing cells were picked. Four weeks old athymic nude mice (BALB/c) were purchased from the Cancer Institute of the Chinese Academy of Medical Science. Experiments with mice were conducted strictly in accordance with a protocol approved by the Administrate Panel on Laboratory Animal Care of China Medical University. Each nude mouse was subcutaneously injected with 6×10^5 cells in the right flank area for subcutaneous implantation. Tumors were measured every seven days and calculated according to the formula: volume (mm^3)=length×width^2/2. The mice were sacrificed and the tumors were isolated after 60 days. For orthotopic inoculations (cells were injected into the right striatum of nude mice), the number of surviving nude mice was

registered and survival analysis was performed using Kaplan-Meier survival curve. Lastly, we detected VM in vivo mice xenograft model by CD34-PAS.
